# Phylogenetic analysis of the complete mitochondrial genome of the white peacock butterfly *Anartia jatrophae saturata* (Insecta: Lepidoptera: Nymphalidae)

**DOI:** 10.1080/23802359.2020.1832929

**Published:** 2020-11-06

**Authors:** Josephine E. Payment, Jeffrey M. Marcus, Melanie M. L. Lalonde

**Affiliations:** Department of Biological Sciences, University of Manitoba, Winnipeg, Canada

**Keywords:** Illumina sequencing, mitogenomics, Nymphalidae, Tribe Victorini, *Anartia*

## Abstract

The white peacock butterfly *Anartia jatrophae saturata* Staudinger, 1884 (Nymphalidae: Nymphalinae: Victorini), lives in the neotropics. Genome skimming with Illumina sequencing of *A. jatrophae saturata* allowed the assembly of a complete circular mitogenome of 15,297 bp, consisting of 81.4% AT nucleotides, 22 tRNAs, 13 protein-coding genes, two rRNAs, and a control region. *Anartia jatrophae COX1* features an atypical start codon (CGA); *ATP6, COX1, ND1, ND4*, *ND4L, ND5,* and *ND6* exhibit incomplete stop codons completed in the mRNA by the addition of 3′ A residues. Contrary to previous phylogenetic hypotheses, phylogenetic reconstruction places *A. jatrophae* as sister to nymphalid tribe Nymphalini.

The white peacock butterfly, *Anartia jatrophae saturata,* Staudinger, 1884, is found in open and disturbed environments as well as secondary forests throughout the Greater Antilles and Florida (Turner and Turland 2017; Pfeiler et al. 2020). *Anartia jatrophae* is a geographically widespread Neotropical species that is known for its territorial behavior (Lederhouse et al. 1992; Blum et al. 2003). The subspecies, *A. jatrophae saturata,* exhibits pronounced sexual dimorphism as well as brightly colored and faded phenotypes, which were once thought to be the result of seasonal variation, but have since been seen throughout the year (Munroe 1942; Turner and Turland 2017). Here we report the complete mitochondrial genome sequence of *A. jatrophae saturata* (Genbank MT712074) from specimen Anja2017.1, collected in the Dominican Republic (GPS 18.736 N, 70.163 W) in December 2017 that has been pinned, spread, and deposited in the Wallis Roughley Museum of Entomology, University of Manitoba (voucher WRME0507733).

DNA was prepared (McCullagh and Marcus 2015) and sequenced by Illumina NovaSeq6000 (San Diego, California) (Marcus 2018). The sequencing library was prepared using NEBNext Ultra II DNA Library Prep Kit for Illumina (New England Biolabs, Ipswich, Massachusetts). The mitogenome of *A. jatrophae saturata* was assembled by Geneious 10.1.2 from 11,195,835 paired 150 bp reads (Genbank SRA accession PRJNA65761) using a *Junonia stygia* (Lepidoptera: Nymphalidae) reference mitogenome (MN623383) (Living Prairie Mitogenomics Consortium 2020). Annotation was in reference to the *J. stygia* mitogenome. The *A. jatrophae saturata* nuclear rRNA repeat (Genbank MT742579) was also assembled and annotated using a *J. stygia* reference sequence.

The *A. jatrophae saturata* circular 15,297 bp mitogenome assembly was composed of 35,004 paired reads with nucleotide composition: 39.1% A, 11.1% C, 7.5% G, and 42.3% T. The gene composition and order in *A. jatrophae saturata* is identical to all known butterfly genomes (Cao et al. 2012; McCullagh and Marcus 2015). *Anartia jatrophae saturata COX1* features an atypical CGA start codon as in many other insects (Liao et al. 2010). The mitogenome contains five protein-coding genes (*COX1, ND1, ND4, ND5, ND6*) with single-nucleotide (T) stop codons, and two protein-coding genes (*ATP6, ND4L*) with two-nucleotide (TA) stop codons completed by post-transcriptional addition of 3′ A residues. The locations and structures of tRNAs were determined using ARWEN v.1.2 (Laslett and Canback 2008). tRNAs have typical cloverleaf secondary structures except for trnS (AGN) where the dihydrouridine arm is replaced by a loop, while the mitochondrial rRNAs and control region are typical for Lepidoptera (McCullagh and Marcus 2015).

We reconstructed a phylogeny using complete mitogenomes from *A. jatrophae saturata* and 41 additional mitogenomes from subfamily Nymphalinae (Lalonde and Marcus 2019a, 2019b; Chen et al. 2020; Alexiuk et al. 2020a, 2020b; Hamilton et al. 2020; Lalonde and Marcus 2020; Payment et al. 2020). Mitogenome sequences were aligned in CLUSTAL Omega (Sievers et al. 2011) and analyzed by parsimony and maximum likelihood (model selected by jModeltest 2.1.7 (Darriba et al. 2012) and likelihood ratio test (Huelsenbeck and Rannala 1997)) in PAUP* 4.0 b/4.0d78 (Swofford 2002) (Figure 1). Previous phylogenetic analyses based on smaller sequence data sets have placed *A. jatrophae* within the nymphalid tribe Victorini (Blum et al. 2003; Wahlberg et al. 2005), and have placed Victorini as sister to either tribe Junonini (Wahlberg et al. 2005; Kodandaramaiah and Wahlberg 2007) or to species that have often been assigned to tribe Kallimini (Wahlberg et al. 2009). Tribe Kallimini is not monophyletic in our analysis. Contrary to these earlier phylogenetic hypotheses, we have found that *A. jatrophae saturata*, the only representative of nymphalid tribe Victorini currently with a sequenced mitogenome, is sister to a clade of mitogenomes from tribe Nymphalini.

**Figure 1.**
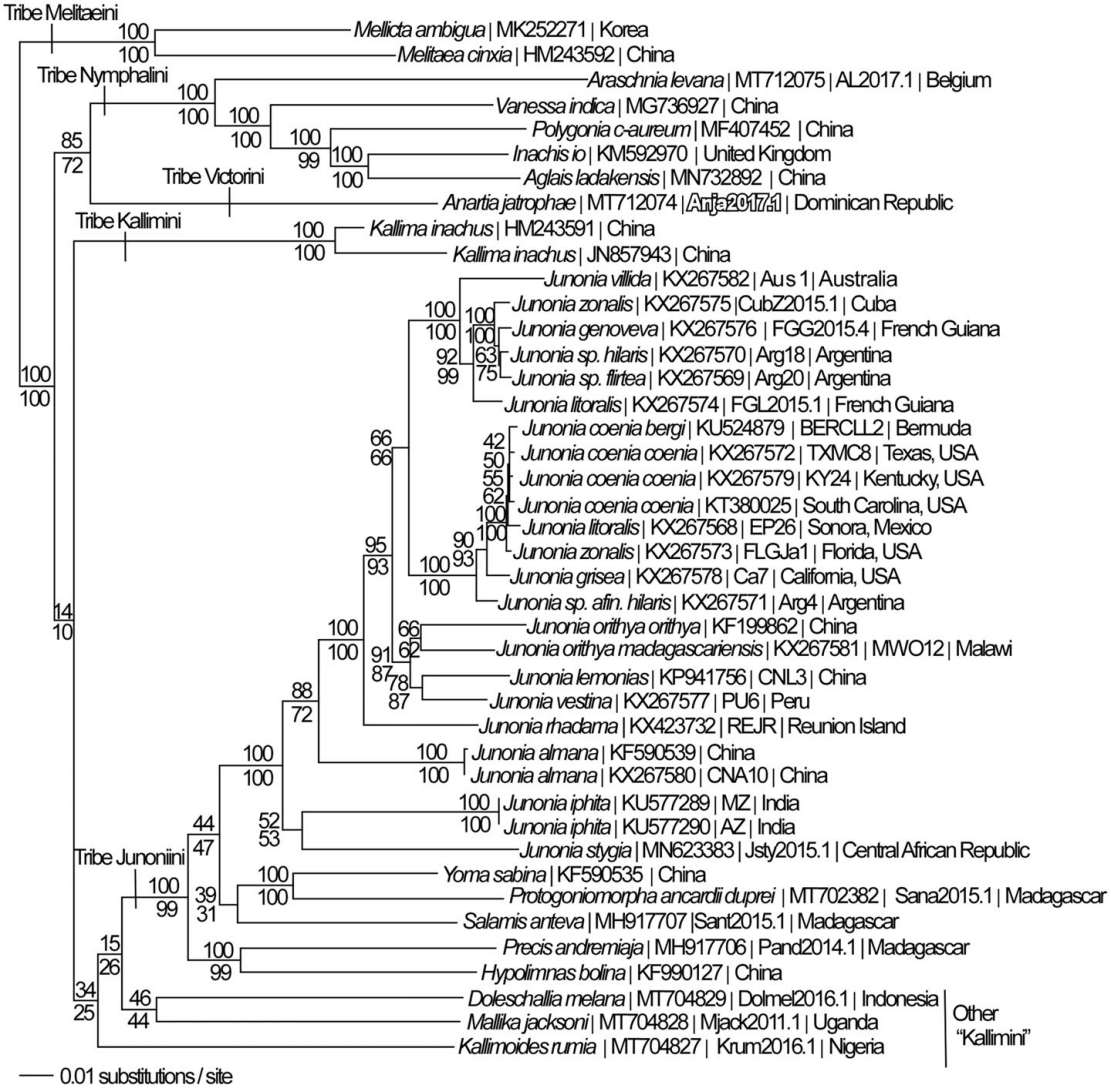
Maximum likelihood phylogeny (GTR + G model, G = 0.2330, likelihood score 117762.66543) of *Anartia jatrophae saturata* (tribe Victorini), 29 mitogenomes from tribe Junonini, 5 from Kallimini, 5 from Nymphalini and 2 outgroup from tribe Melitaeini in subfamily Nymphalinae based on 1 million random addition heuristic search replicates (with tree bisection and reconnection). One million maximum parsimony heuristic search replicates produced 16 trees (parsimony score 20,698 steps) which differ from one another only by the arrangement of *Junonia coenia* mitogenomes and one of which has an identical tree topology to the maximum likelihood tree depicted here. Numbers above each node are maximum likelihood bootstrap values and numbers below each node are maximum parsimony bootstrap values (each from 1 million random fast addition search replicates).

## Data Availability

The data that support the findings of this study are openly available in GenBank of NCBI at https://www.ncbi.nlm.nih.gov, reference numbers MT712074, MT742579, and PRJNA657614.

## References

[CIT0001] Alexiuk MR, Marcus JM, Lalonde MML. 2020a. The complete mitochondrial genome and phylogenetic analysis of the European map butterfly *Araschnia levana* (Insecta: Lepidoptera: Nymphalidae). Mitochondrial DNA B Resour. 10.1080/23802359.2020.1810163 PMC7782646 33458126

[CIT0002] Alexiuk MR, Marcus JM, Lalonde MML. 2020b. The complete mitochondrial genome of the Jackson’s Leaf butterfly *Mallika jacksoni* (Insecta: Lepidoptera: Nymphalidae). Mitochondrial DNA B Resour. 10.1080/23802359.2020.1814885 PMC7782007 33458145

[CIT0003] Blum MJ, Bermingham E, Dasmahapatra K. 2003. A molecular phylogeny of the neotropical butterfly genus *Anartia* (Lepidoptera: Nymphalidae). Mol Phylogen Evol. 26(1):46–55. 10.1016/s1055-7903(02)00291-9 12470937

[CIT0004] Cao YQ, Ma C, Chen JY, Yang DR. 2012. The complete mitochondrial genomes of two ghost moths, *Thitarodes renzhiensis* and *Thitarodes yunnanensis*: the ancestral gene arrangement in Lepidoptera. BMC Genomics. 13:276. 22726496 10.1186/1471-2164-13-276 PMC3463433

[CIT0005] Chen K, Si C, Pan Z, Hao J. 2020. The complete mitochondrial genome of *Aglais ladakensis* (Lepidoptera: Nymphalidae: Nymphalinae. Mitochondrial DNA B Resour. 5(1):639–641. 33366682 10.1080/23802359.2019.1711224 PMC7748854

[CIT0006] Darriba D, Taboada GL, Doallo R, Posada D. 2012. jModelTest 2: more models, new heuristics and parallel computing. Nat Methods. 9(8):772 10.1038/nmeth.2109 PMC4594756 22847109

[CIT0007] Hamilton RV, Marcus JM, Lalonde MML. 2020. The complete mitochondrial genome of the black dead leaf Butterfly *Doleschallia melana *(Insecta: Lepidoptera: Nymphalidae). Mitochondrial DNA B Resour. 10.1080/23802359.2020.1814885 PMC7782007 33458145

[CIT0008] Huelsenbeck JP, Rannala B. 1997. Phylogenetic methods come of age: testing hypotheses in an evolutionary context. Science. 276(5310):227–232. 9092465 10.1126/science.276.5310.227

[CIT0009] Kodandaramaiah U, Wahlberg N. 2007. Out-of-Africa origin and dispersal-mediated diversification of the butterfly genus *Junonia* (Nymphalidae: Nymphalinae). J Evol Biol. 20(6):2181–2191. 17887973 10.1111/j.1420-9101.2007.01425.x

[CIT0010] Lalonde MML, Marcus JM. 2019a. The complete mitochondrial genome of the Madagascar mother-of-pearl butterfly *Salamis anteva* (Insecta: Lepidoptera: NympChalidae). Mitochondrial DNA B Resour. 4(1):296–298.

[CIT0011] Lalonde MML, Marcus JM. 2019b. The complete mitochondrial genome of the Madagascar banded commodore butterfly *Precis andremiaja* (Insecta: Lepidoptera: Nymphalidae). Mitochondrial DNA B Resour. 4(1):277–279.

[CIT0012] Lalonde MML, Marcus JM. 2020. The complete mitochondrial genome of the Malagasy clouded mother-of-pearl butterfly *Protogoniamorpha ancardii duprei* (Insecta: Lepidoptera: Nymphalidae). Mitochondrial DNA B Resour. 10.1080/23802359.2020.1810156 PMC7782682 33458125

[CIT0013] Laslett D, Canback B. 2008. ARWEN: a program to detect tRNA genes in metazoan mitochondrial nucleotide sequences. Bioinformatics. 24(2):172–175. 18033792 10.1093/bioinformatics/btm573

[CIT0014] Lederhouse RC, Codella SG, Grossmueller DW, Maccarone AD. 1992. Host plant-based territoriality in the white peacock butterfly *Anartia jatrophae* (Lepidoptera: Nymphalidae). J Insect Behav. 5(6):721–728.

[CIT0015] Liao F, Wang L, Wu S, Li Y-P, Zhao L, Huang G-M, Niu C-J, Liu Y-Q, Li M-G. 2010. The complete mitochondrial genome of the fall webworm, *Hyphantria cunea* (Lepidoptera: Arctiidae). Int J Biol Sci. 6(2):172–186. 20376208 10.7150/ijbs.6.172 PMC2850540

[CIT0016] Living Prairie Mitogenomics Consortium. 2020. The complete mitochondrial genome of the brown pansy butterfly, *Junonia stygia* (Aurivillius, 1894), (Insecta: Lepidoptera: Nymphalidae). Mitochondrial DNA B Resour. 5:41–43. 10.1080/23802359.2019.1693921 PMC7720999 33366413

[CIT0017] Marcus JM. 2018. Our love-hate relationship with DNA barcodes, the Y2K problem, and the search for next generation barcodes. AIMS Genetics. 5(1):1–23. 31435510 10.3934/genet.2018.1.1 PMC6690253

[CIT0018] McCullagh BS, Marcus JM. 2015. The complete mitochondrional genome of Lemon Pansy, *Junonia lemonias* (Lepidoptera: Nymphalidae: Nymphalinae). J Asia-Pacific Ent. 18(4):749–755.

[CIT0019] Munroe EG. 1942. The Caribbean races of *Anartia jatrophae* Johansson (Lepidoptera: Nymphalidae). Am Mus Novit. 1179:1–4.

[CIT0020] Payment JE, Marcus JM, Lalonde MML. 2020. The complete mitochondrial genome of the African leaf butterfly *Kallimoides rumia* (Insecta: Lepidoptera: Nymphalidae). Mitochondrial DNA B Resour.

[CIT0021] Pfeiler E, Nazario-Yepiz NO, Hernández-Cervantes PL, Markow TA. 2020. Mitochondrial DNA barcodes provide insight into the phylogeography and subspecies controversy in the widespread Neotropical white peacock butterfly, Anartia jatrophae (Nymphalidae: Nymphalinae). Biol J Linn Soc.

[CIT0022] Sievers F, Wilm A, Dineen D, Gibson TJ, Karplus K, Li W, Lopez R, McWilliam H, Remmert M, Söding J, et al. 2011. Fast, scalable generation of high-quality protein multiple sequence alignments using Clustal Omega. Mol Syst Biol. 7:539 21988835 10.1038/msb.2011.75 PMC3261699

[CIT0023] Swofford DL. 2002. PAUP*. Phylogenetic analysis using parsimony (*and other methods). Version 4. Sunderland (MA): Sinauer Associates.

[CIT0024] Turner T, Turland V. 2017. Discovering Jamaican butterflies and their relationships around the Caribbean. Safety Habor (FL): Caribbean Wildlife Publications.

[CIT0025] Wahlberg N, Brower AVZ, Nylin S. 2005. Phylogenetic relationships and historical biogeography of tribes and genera in the subfamily Nymphalinae (Lepidoptera: Nymphalidae). Biol J Linn Soc. 86(2):227–251.

[CIT0026] Wahlberg N, Leneveu J, Kodandaramaiah U, Peña C, Nylin S, Freitas AVL, Brower AVZ. 2009. Nymphalid butterflies diversify following near demise at the Cretaceous/Tertiary boundary. Proc Biol Sci. 276(1677):4295–4302. 19793750 10.1098/rspb.2009.1303 PMC2817107

